# Mechanical Performance of Adhesive Connections in Structural Applications

**DOI:** 10.3390/ma16227066

**Published:** 2023-11-07

**Authors:** Marco Lamberti, Aurélien Maurel-Pantel, Frédéric Lebon

**Affiliations:** 1 Aix-Marseille University, CNRS, Centrale Marseille, LMA, 13453 Marseille, France; 2 ENEA, Brasimone Research Center, 40032 Camugnano, Italy

**Keywords:** adhesive bonding, damage, porosity, Kachanov–Sevostianov’s material

## Abstract

Adhesive bonding is an excellent candidate for realising connections for secondary structures in structural applications such as offshore wind turbines and installations, avoiding the risk and associated welding problems. The strength of the adhesive layer is an important parameter to consider in the design process it being lower than the strength capacity of the bonding material. The presence of defects in the adhesive materials undoubtedly influences the mechanical behaviour of bonded composite structures. More specifically, the reduction in strength is more pronounced as the presence of defects (voids) increases. For this reason, a correct evaluation of the presence of defects, which can be translated into damage parameters, has become essential in predicting the actual behaviour of the bonded joints under different external loading conditions. In this paper, an extensive experimental programme has been carried out on adhesively bonded connections subjected to Mode I and Mode II loading conditions in order to characterise the mechanical properties of a commercial epoxy resin and to define the damage parameters. The initial damage parameters of the adhesive layer have been identified according to the Kachanov–Sevostianov material definition, which is able to take into account the presence of diffuse initial cracking.

## 1. Introduction

In the last two decades, the use of adhesive materials in the field of mechanical and civil engineering has grown exponentially due to their capacity to easily and quickly connect several types of materials to each other such as metals, composite, concrete and masonry.

The use of bonding techniques in various industries has increased significantly due to the growing demand for the design of lightweight structures in the mechanical field, such as aircraft and vehicle frames. For this reason, the use of adhesive bonding to join advanced lightweight materials that are dissimilar, coated, and difficult to weld have been widely studied in recent years [1]. 

Although bonding has been used as a traditional joining method for many centuries, it is only in the last seventy years that the scientific results and the technology of the bonding technique have advanced significantly [2,3,4,5,6,7]. In addition to civil engineering, the adhesive bonding technique has been increasingly used in structural strengthening and reinforcement of concrete elements by adding FRP sheets, both in fully composite structures such as pedestrian bridges and in buildings where pultruded profiles have been matched to form complex and structured cross-sections [8]. Furthermore, these types of joints are particularly suitable for the realization of secondary structures such as parapets, stairs and railings in various types of structures such as buildings, cooling towers and offshore installations. 

Among the factors which have limited the spread and development of adhesive connections for marine and offshore structures, there is the long-term durability of joints in critical environments.

Nowadays, in offshore installations, most connections are made using the welding technique. However, the welding technique does not represent the optimal solution for safety and building technique reasons. Avoiding the presence of high welding temperatures leads to safer construction in marine environments. In addition, it will positively contribute to the preservation of and improvement in the quality of the environment by reducing the amount of welding slag created.

Adhesive bonding in the marine environment for offshore applications is still very much in its infancy despite some successes. However, it is still needed to establish this joining process as a standard process considering the design, fabrication, and modification of offshore structures. 

It is important to emphasize that the choice of thickness geometry must derive from on-site feasibility assessments, considering that thin and uniform adhesive thicknesses are easily made in a specialized laboratory using skilled workers, otherwise it becomes difficult to make them on site.

For these reasons, the scientific and industrial communities have become interested in providing tools to describe and simulate the behaviour of adhesively bonded joints.

The mechanical behaviour of an adhesive joint is influenced not only by the geometry of the joint, but also by various boundary conditions.

Several approaches and theories have been formulated in the literature to describe material characteristics to investigate different types of applications using analytical, mechanical, or finite element analyses. Among them, damage modelling is increasingly used to simulate debonding processes and fractures in adhesive connections.

One of the most important characteristics is undoubtedly the stiffness of the adhesive layer, which, if properly defined, allows a realistic evaluation of the displacements exhibited after the application of loads that could act during the life of the structure.

Damage modelling techniques are distinguished into local or continuous approaches. In the first, the continuous approach, damage is implemented over a finite region, while in the second, the local approach, damage is located to zero-volume lines leading it to be referred as the cohesive zone model [9].

The cohesive zone model [10] has received considerable attention over the past two decades and has been used to predict interlaminar failure of composite materials. Fractures in bonded materials particularly affect the machined zone in front of the macrocrack tip, where microcracks or cavities form, grow and coalesce. This process region can be modelled by assuming that the material along the crack path follows the established tensile separation laws of an appropriate cohesive region model. There are a large number of cohesion laws in the literature, ranging from exponential to trapezoidal laws.

One of the earliest theories of the elastic contact model for flat metal surfaces was formulated by Greenwood and Williamson [11]. 

The model proposed was based on the existence of elastic contact hardness, a composite quantity that is a function of the elastic properties and topography, considering a statistical distribution of asperities that do not interact with each other.

Subsequently, Yoshioka and Scholz [12] developed a theory for predicting the behaviour of contacting surfaces focused on micromechanics under elastic and non-slip conditions, opening up a new way of understanding the behaviour of contacting surfaces.

A few years later, Sherif and Kossa [13], using the theories of Greenwood and Williamson [11], carried out a theoretical analysis to calculate the normal and tangential contact stiffnesses between two elastic flat surfaces, giving an interpretation of the experimental results obtained founded on the evaluation of the natural frequencies at the contact region. Following the same strategy, Krolikowski and Szczepek [14], based on the Green–Wood–Williams model and the Hertz–Mindlin theory [15], provided an analytical description of the normal and tangential contact stiffness between rough surfaces with spherical properties. Contact stiffness has also been measured using an ultrasonic method focused on the measure of the reflection coefficient of ultrasonic waves at the interface.

The definition of contact stiffness has been carried out by several experimental studies that can be found in the literature.

In addition to the cases mentioned above [13,14], Gonzalez-Valadez et al. [16] proposed the use of a simple spring model influenced by the amount, shape and distribution of the contact asperities, relating the interfacial stiffness to the reflection of ultrasound obtained in a rough contact. 

Finally, a new approach has been proposed by Kachanov et al. [17]. 

The Kachanov theory consists of considering the presence of initial cracks in the interior of an adhesive material. The main assumptions of the microcracked adhesive are based on the absence of interaction among the several cracks, constant stress vector along the crack and finally the absence of effect due to the presence of the crack edge in the stress field. Furthermore, the peculiarity of this model is that it considers some of the most important variabilities of the adhesive, such as thickness variation, porosity and initial damage [18,19].

The Kachanov-type model has previously been successfully applied to aluminium foam alloy [18], composite materials [19] and also other types of structures.

The accuracy of this approach, which is a function of the density of the cracks, is satisfactory up to fairly small distances between the cracks. The distances between the cracks is much smaller than their width. For linear cracks, Kachanov’s model includes a global parameter indicated as crack density, which is attributable to the number and length of all cracks.

In this work, an extensive experimental programme was carried out to determine the properties of the undamaged material. The experimental programme consists of static tensile tests performed under Mode I and Mode II loading conditions on bonded specimens using an Arcan-modified apparatus and double lap shear-bonded joints. 

The bonded joints were realised with different sizes of thickness and surface area of the adhesive layer in order to provide a better comprehension of the damage parameters. Finally, an imperfect interface model, obtained thanks to the homogenisation technique and the asymptotic approach, was used to reproduce the global response of the adhesive joints in Mode I and II loading conditions, using the initial damage parameters evaluated experimentally.

Since it is essential that the adhesive connections must be able to guarantee long-term properties and sufficient mechanical strength in order to propose reliable solutions, the effects of the aging conditions will be investigated as a perspective of the present investigation.

## 2. Experimental Program

To characterise the mechanical behaviour of adhesive connections under normal and tangential forces, two types of specimens were produced and tested: cylindrical and double lap shear joints.

In addition, several adhesive thicknesses and diameters for the cylindrical specimens were experimentally tested under static loading conditions in order to evaluate the variation in damage parameters as a function of adhesive volume. 

The adhesive used in the current investigation is available on the market and is named Sicomin Isobond SR 5030/SD 503x [20]. Specifically, Sicomin Isobond is a two-component epoxy paste designed for structural bonding and fillet joints, with high mechanical strength and high thixotropy for good behaviour on vertical surfaces. The maximum strength of the adhesive is reached after a curing time of 24 h at 23 °C or 10 h at 70 °C.

The mechanical properties declared by the manufacturer are summarized in Table 1.

### 2.1. Cylindrical Adhesive Joints

Cylindrical aluminium specimens were used to make the adhesive joints. The mechanical properties of the aluminium material are provided in Table 2. Each specimen has a straight surface which allows a homogeneous adhesive layer to be produced at the interface.

**Figure 1:**
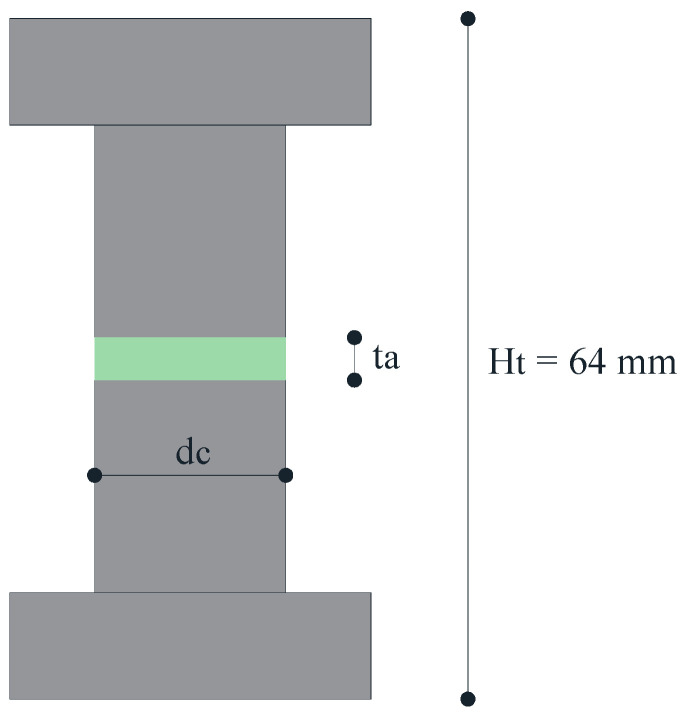
Dimension of cylinder adhesive connections.

The cylindrical adhesive samples are realized using a steel device made by an upper and lower horizontal element connected to each other by means of two vertical columns; in this way, a constant total height of the adhesive layer is performed. The total height, *Ht*, of 64 mm is due to the height of two half-specimens plus the adhesive thickness of the adhesive. In more detail, three different thicknesses, *ta*, are tested: 1, 2.5, and 5 mm and three-cylinder diameters considered, *dc*: 18, 14 and 10 mm. Further geometrical details are reported in Figure 1. The samples were cured at room temperature for 24 h.

To ensure the effectiveness of the bonding, the surfaces were well-cleaned using acetone. Surfaces may be contaminated with dust or micro-particles and may have poorly adhering surface layers, which affects the effectiveness of bonding and may lead to premature failure.

### 2.2. Double Lap Shear Joints

**Figure 2:**
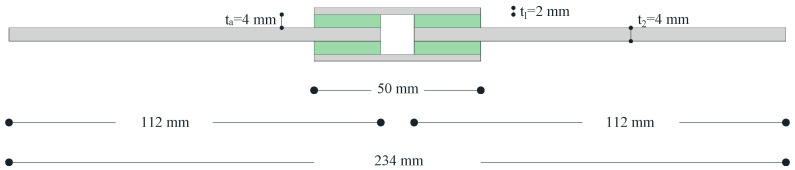
Double lap shear adhesive joint dimension.

A total of 6 double lap shear joints were manufactured to investigate the shear strength of the epoxy adhesive. The specimens were realized in accordance with the standard code ASTM D3528-96 [21] using rectangular S275 steel elements. More specifically, the specimens were made up of two rectangular plates of 112 mm, 26 mm wide and 4 mm thick, and two other rectangular plates of 50 mm, 26 mm wide and 2 mm thick (some details in Figure 2). The steel elements were bonded together with four 20 × 26 mm rectangular adhesive layers.

The mechanical properties of the steel plate are reported in Table 3.

**Figure 3:**
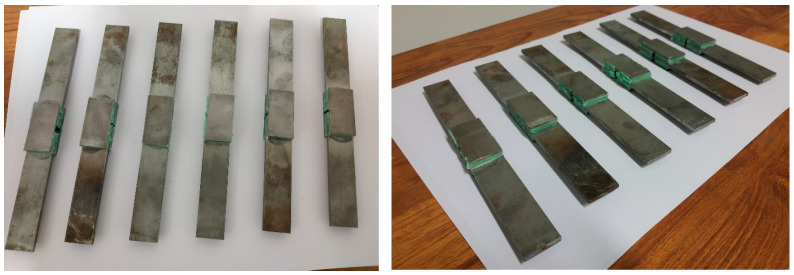
Double lap shear adhesive specimens.

Figure 3 shows the double lap shear adhesive specimens realized.

## 3. Experimental Tests

All the tests were performed at the Laboratory of Mechanics and Acoustics in Marseille using the universal testing machine characterized by a load capacity of 100 kN. 

As is well known in the literature, in fracture mechanics which are concerned with the study of crack propagation in materials, the force is divided into its components. This process leads to the definition of the following two modes: Mode I, also known as the “opening mode” where a tensile stress is applied perpendicular to the plane of the crack, and Mode II, also known as the “sliding mode” where a shear stress is applied parallel to the plane of the crack.

In the current investigation, the specimens were subjected to both Mode I and II loading conditions. 

The experimental set-up and results are described and discussed in the following sections.

### 3.1. Cylindrical Adhesive Joints in Mode I

A total of 37 cylindrical adhesive joints with different surfaces and volumes of adhesive layer have been programmed and realized. 

**Figure 4:**
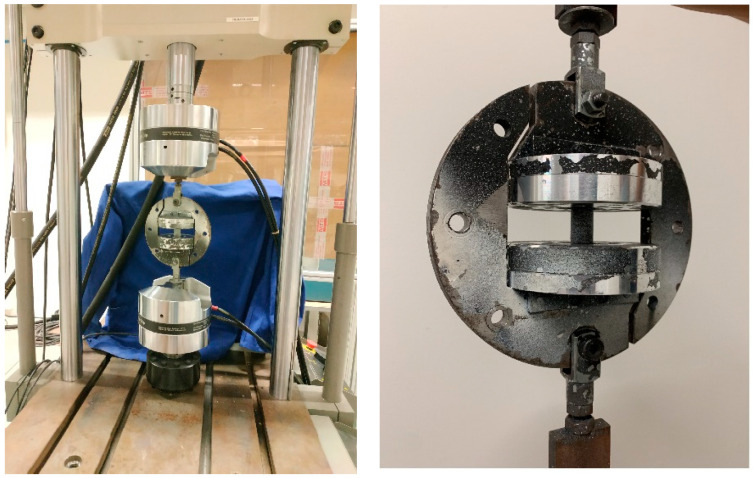
Experimental set-up of adhesive connections subject to Mode I loading by means of an Arcan-modified device.

The cylindrical adhesive joints were subjected to a vertical displacement by means of an Arcan-modified device at a rate of 1 mm/min. The experimental test set-up is depicted in Figure 4.

### 3.2. Double Lap Shear Joints in Mode II

**Figure 5:**
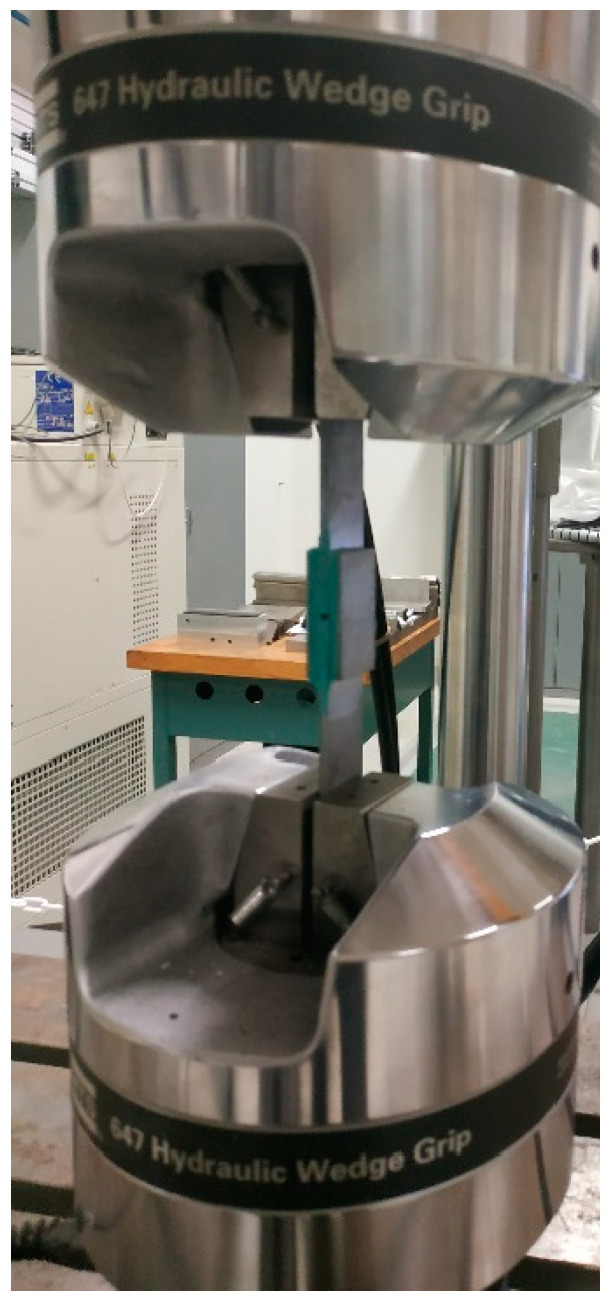
Experimental set-up of double lap shear tests.

The double lap shear joints were placed directly in the clamps of the universal testing machine, as shown in Figure 5. All the specimens were tested in displacement control at a rate of 1 mm/min.

## 4. Experimental Test Results

In this section, the experimental results are analyzed and discussed for both aluminium cylindrical and double lap shear adhesive joints tested in Mode I and II loading conditions. 

### 4.1. Cylindrical Adhesive Joints Test Results

The experimental results of cylindrical adhesive joints are evaluated in this section. As mentioned above, in the current investigation, several diameters were considered equal to 18, 14 and 10 mm, respectively, and for each of them the adhesive thicknesses equal to 1, 2.5 and 5 mm have been investigated. 

The experimental data are summarized in the following tables in terms of ultimate force, F_u_, ultimate stress, σ_u_, corresponding displacement u_max_, and global stiffness K.

In more detail, the experimental results of cylindrical adhesive joints characterized by a diameter of 18 mm at different adhesive thicknesses are reported in Table 4. 

In Table 5 and Table 6, the experimental data of adhesive joints of diameter equal to 14 and 10 mm at different adhesive thicknesses are reported, respectively.

It is important to note that the choice of high thicknesses is due to their feasibility on site by workers for the realization of adhesive connection for secondary structures in civil and mechanical engineering construction. However, the value of standard deviation reported in Table 4, Table 5 and Table 6 can be explained by the presence of initial defects inside the adhesive layer. 

**Figure 6:**
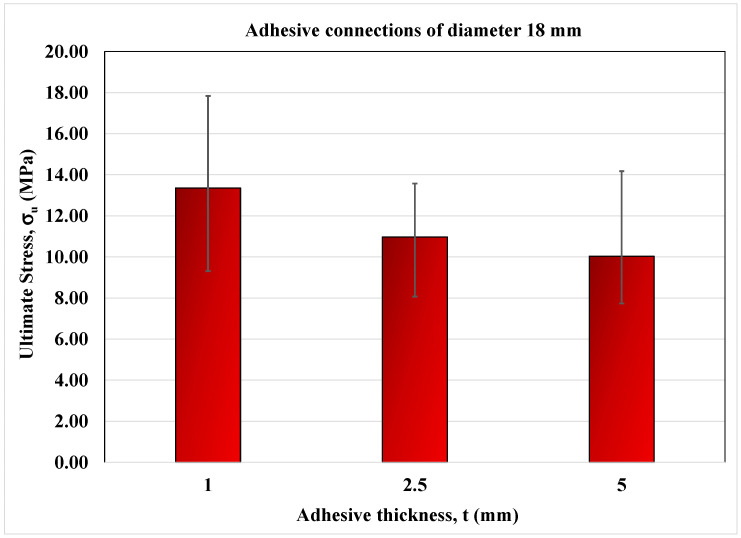
Bar-chart of adhesive connections of diameter equal to 18 mm.

**Figure 7:**
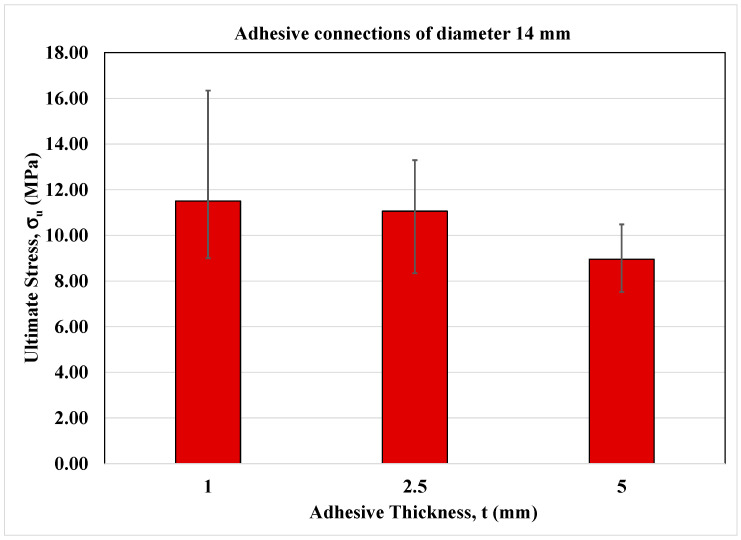
Bar-chart of adhesive connections of diameter equal to 14 mm.

**Figure 8:**
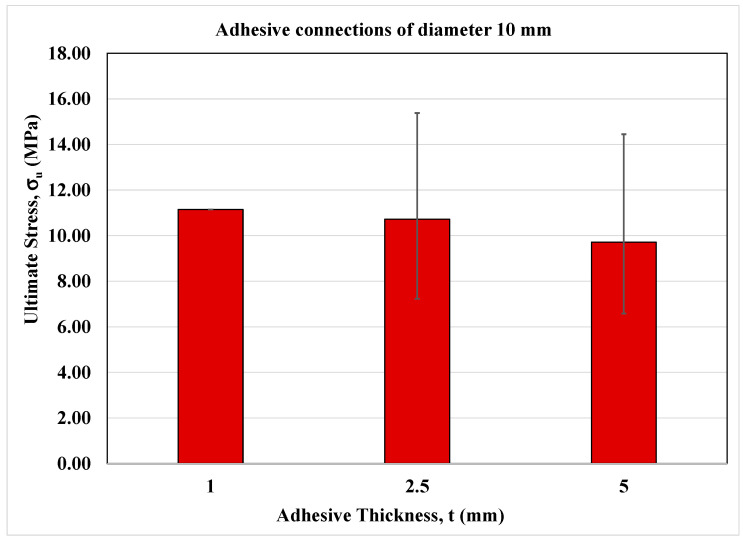
Bar-chart of adhesive connections of diameter equal to 10 mm.

On the other hand, Figure 6, Figure 7 and Figure 8 show the bar charts in terms of ultimate normal stress for the adhesive joints under monotonic loading conditions for each cylinder diameter and adhesive thickness.

As can be seen, the strength is higher at lower adhesive thicknesses and similar between the thicknesses of 2.5 and 5 mm.

**Figure 9:**
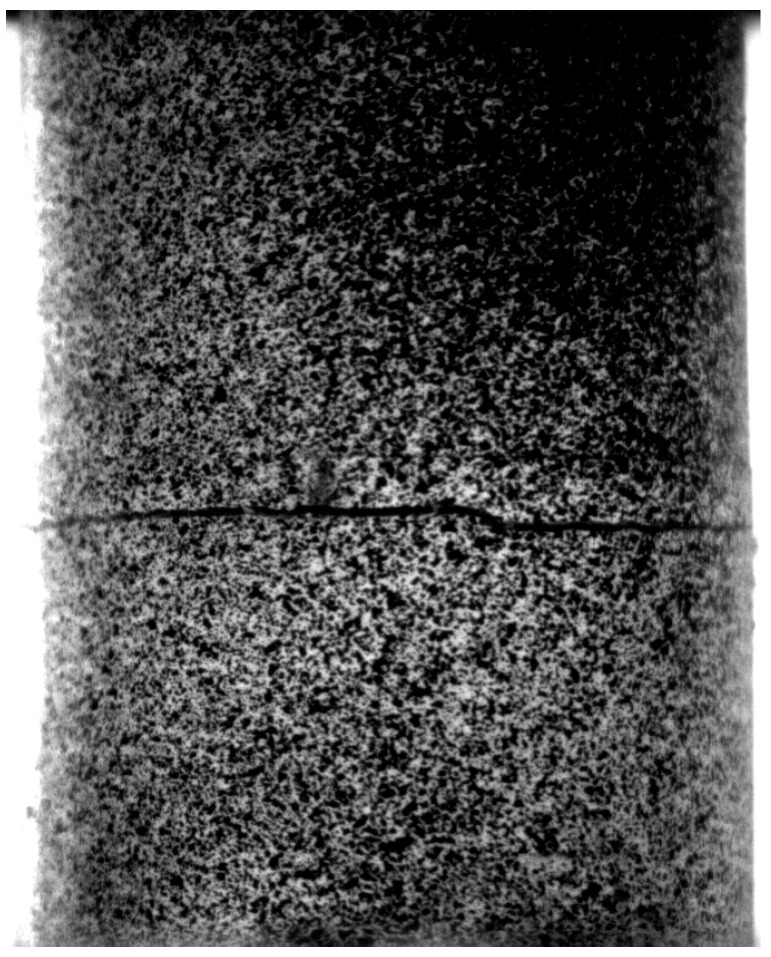
Cohesive failure recorded by the camera for the specimen D18T2.5#2.

For each specimen, the failure has occurred after the initiation of a crack in the adhesive layer and its instantaneous propagation, resulting the separation of the bonded metallic elements (see Figure 9). After the failure, some of the adhesive layer remains on the two cylindrical surfaces (cohesive failure).

### 4.2. Double Lap Shear Test Results 

The experimental results of double lap shear adhesive tests in terms of ultimate force, F_u_; average shear stress, τ_m_; displacement at failure, u_max_; and global stiffness, K, are summarized in Table 7.

**Figure 10:**
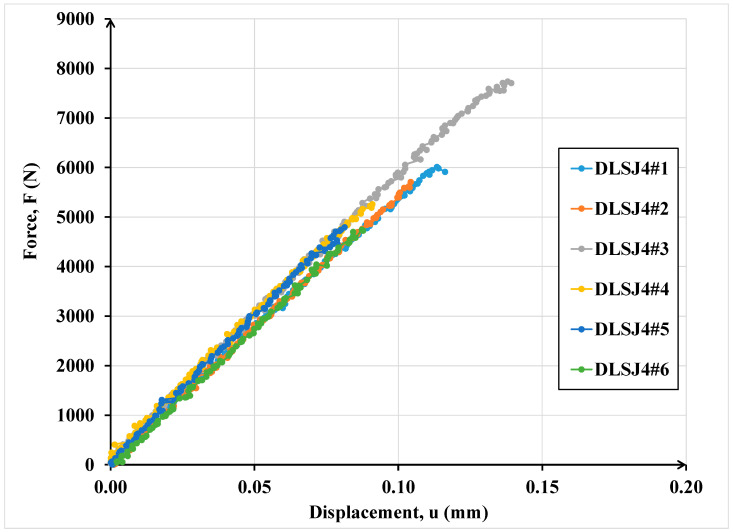
Global response of double lap shear tests.

Figure 10 shows the global mechanical response in terms of force versus displacement of the double lap shear adhesive joints.

The failure occurs after the initiation of a crack in the adhesive layer and the consequent instantaneous propagation, which leads to the separation of the bonded metallic adherents.

**Figure 11:**
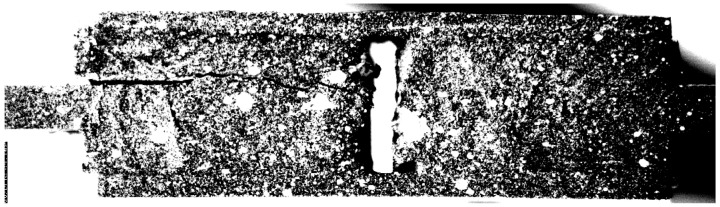
Detailed view of the failed adhesive area for the specimen DLSJ4#3.

Figure 11 shows the picture recorded by the camera at the failure instant for the specimen DLSJ4#3. 

## 5. Damage Parameters

The results of the experimental investigations made it possible to evaluate the initial damage properties of a material defined according to Kachanov–Sevostianov’s material theory.

In Equations (1) and (2), the stiffness in Mode I and II, respectively, is defined by the function of the adhesive Young’s Moduli, *E*, in the normal and tangential direction; the Poisson ratio, ν; the geometric dimension of the adhesive, *S*; and initial damage length, *l*_0_. Note that the stiffness is directly dependent on the length *l*_0_.

In this approach, the crack density *ρ*(*l*_0_) is defined in Equation (3) [17,18,19]. In more detail, the crack density can be evaluated as the ratio between the cubic length of the crack *l_0_* and the elementary volume in this way is able to describe the material at the microscale. Note that the crack density is therefore inversely proportional to the thickness of the adhesive interface:

(1) $$K_{N}\left(l_{0}\right)=\frac{3E_{N}S}{16l_{0}^{3}\left(1-\nu^{2}\right)}$$

(2) $$K_{T}\left(l\right)=\frac{3E_{T}S\left(2-\nu \right)}{32l_{0}^{3}\left(1-\nu^{2}\right)}$$

(3) $$\rho \left(l_{0}\right)=\frac{l_{0}^{3}}{V}$$

Finally, using the experimental results in terms of stiffness in Equations (1) and (2), the initial damage lengths *l*_0_ are calculated according to Kachanov–Sevostianov’s theory for Mode I and II and are summarized in Table 8a,b, respectively.

As highlighted in Table 8, the initial crack length assumes higher values as the adhesive volume increases due to a higher presence of defects or voids. As expected, the initial crack length as a damage parameter is a function of the volume of the adhesive layer.

## 6. Imperfect Interface Model

In this section, the steps are illustrated that led to the formulation of the imperfect interface model. The theoretical model is obtained by homogenization techniques and by asymptotic methods in the context of small perturbation coupling of unilateral contact and damage [18,19,22,23,24,25,26]. 

The approach of the considered damage behaviour is introduced in [24,27]: a thin adhesive interphase is located between two elements (adherents) and is assumed to be a microcracked material undergoing a degradation process. Further details can be found in [26,27].

Each step of the proposed procedure is described below:

(1) The microstructure of the glue layer incorporates multiple families of randomly arranged and distributed microcracks. The family of parallel microcracks is chosen as the only representative of the macroscopic behaviour of the adhesive and is indicated as the equivalent length *l* of the family of microcracks. Furthermore, the direction of the crack is considered to be parallel to the adherent surface.
(2) The actual mechanical properties of theoretical microcracked elements are obtained through the Kachanov-type homogenization mathematical technique [18,19], based on the Eshelby problem. The consequent elastic properties depend on the microcrack density *ρ*, whose three-dimensional formula is $\rho =\frac{l^{3}}{V}$, where *V* is the volume of the representative element. It is emphasized that the equivalent length *l* of a family of microcracks can be characterized experimentally, as illustrated in Section 5.
  The Young’s modulus *E_N_* is defined in the normal direction to the adhesive joint surface and is equal to $\frac{E_{0}}{1+C\rho }$ where *E*_0_ is the initial Young’s modulus and *C* is calculated as $\frac{16(1-v_{0}^{2})}{3}$ where *ν*_0_ is the Poisson’s ratio. Note the subscript 0 indicates the undamaged material.
  The peculiarity of the present model is centred in the definition of the crack density. This crack density changes over the time and therefore represents a damage parameter. Similarly to the crack density *ρ,* the equivalent length *l* is defined by an evolution law. In fact, the time variation of *l* has to be related to a dissipative pseudo-potential *ϕ*, which is given by the sum of quadratic term (rate dependent) and a positively homogeneous functional (rate-independent) [27].
  The dissipative pseudo-potential equation is defined in Equation (4), where *η* indicates a positive viscosity parameter function of the adhesive layer thickness and *I*_B_ denotes the indicator function of a set *B*, in particular $I_{B}=0$ if x∈B and $I_{B}=\infty$ otherwise.
  (4) $$\phi \left(\dot{l}\right)=\frac{1}{2}\eta {\dot{l}}^{2}+I_{]0,\infty [}\left(\dot{l}\right)$$
  Additionally, the indicator function term $I_{]0,\infty [}$ forces the length of cracks to acquire a positive value. This causes the crack length to increase over time, making the adhesive degradation process irreversible.
  Furthermore, damage will only begin when the elastic work is greater than a certain value which depends on the adhesive geometry (thickness) [27].
  It has been proven that Kachanov-type materials are soft materials, this means that, for example, the stiffness of the glue is of the same order of its thickness.
  However, in order to force one-sided contact, which implies non-penetrating conditions during asymptotic expansion, the adhesive is considered a soft material only under tension (Equation (4)).
(3) The homogenised material is employed to implement a thin adhesive interphase. As aforementioned, the interface is located between two elements or adherents. The adhesion between the interface and element’s surface is considered perfect, which means that the continuity of interface separation and of stress vectors is always verified.
(4) Using an appropriate asymptotic expansion [22], due to the small thickness of the glue layer, it is possible to obtain at the limit that the interphase volume of the adhesive is substituted by an interface named *S* of normal unit *n*.
  The equations between the two adherents that link the stress vector *n* and the interface separation [u] across the surface *S* are obtained:
  (5) $$\sigma n=K\left(l\right){\left[u\right]}_{+}+\tau n\;\mathrm{on}\;S$$
  (6) $$\tau \left[u\right].n=0,\;\tau \leq 0,\;\left[u\right].n\geq 0\;\mathrm{on}\;S$$
  (7) $$\bar{\eta }\dot{l}={\left(\bar{\omega }-\frac{1}{2}K_{,l}\left(l\right){\left[u\right]}_{+}.{\left[u\right]}_{+}\right)}_{+}\;\mathrm{on}\;S$$
  For more clarity, the symbol ${\left(\right)}_{,l}$ denotes the partial derivate in *l*, ${\left(\right)}_{+}$ that represent the positive part of a function, i.e., ${\left[u\right]}_{+}$ = $\left[u\right]$ if $\;\left[u\right].n\geq 0$, ${\left[u\right]}_{+}=\left[u\right]-\left[u\right].n$ if $\;\left[u\right].n\leq 0$. The quantity $\bar{\eta }$ is the limit of ηε for ε→0 as well as the limit of ωε (further details on the application of asymptotic expansion can be found in [22]).
  Moreover, the term *K* represents the interface stiffness. As it is possible to note, the interface constitutive law provided in Equations (5)–(7) is a spring-like nonlinear interface model characterized by a nonlinear damage evolution. It is important to emphasize that the interface stiffness *K* remembers the mechanical properties of the initial interphase (mechanical properties, geometry, and damage).

## 7. Finite Element Simulation

The imperfect model presented in Section 6, was implemented in the commercial finite element software, COMSOL Multiphysics 5.6 [28], to verify the reliability of the estimated damage parameters. Both experimental tests on specimens are simulated. Finally, the comparison between numerical and experimental investigation is performed.

### Validation of the Model on Adhesive Tests

In the current model, aluminium and steel substrates assume an isotropic linear elastic behaviour. The adherents’ mechanical properties are reported in Table 2 and Table 3, for aluminium and steel, respectively. 

The interface adhesive model offers the link between the two substrates and implements the mechanical behaviour of the adhesive layer which mechanical properties are collected in Table 1 and by integrating the effect of damage parameters evaluated in Table 8a,b. Thanks to the presence of a symmetry plane only a quarter of the cylindrical specimen and only half part of the double lap shear joint have been modelled.

**Figure 12:**
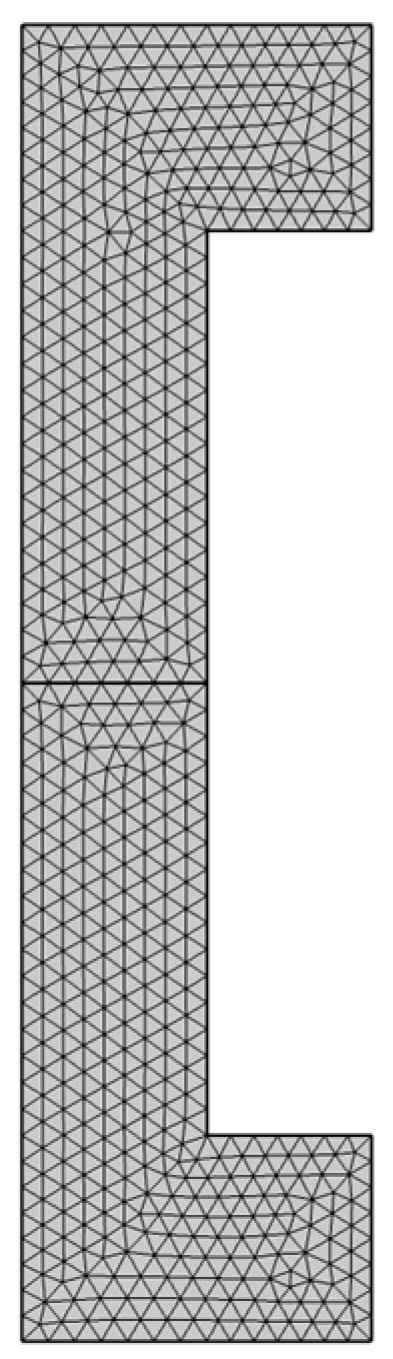
Mesh details of cylindrical joints.

**Figure 13:**
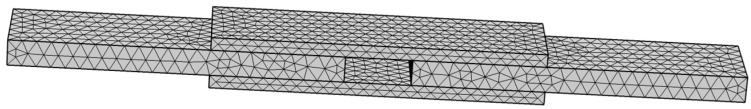
Mesh details of double lap shear joints.

The mesh of the specimen is depicted in Figure 12 and Figure 13.

After a mesh sensitivity analysis involved on the elastic response of the adhesive connection, a fine mesh size (minimal 0.1 mm) of elements is implemented. In more detail, for meshing the entire cylindrical geometry, triangular elements were used while for what concerns the double lap shear joint, tetrahedral elements were employed. 

Boundary conditions correspond to the experimental set-up for both tests: the specimen is embedded at one surface and on the opposite extremity a displacement along the vertical axis is applied. 

The value of normal and tangential stiffnesses have been evaluated by means of the experimental data extrapolated by the global mechanical response and reported in Table 4 and Table 7.

**Figure 14:**
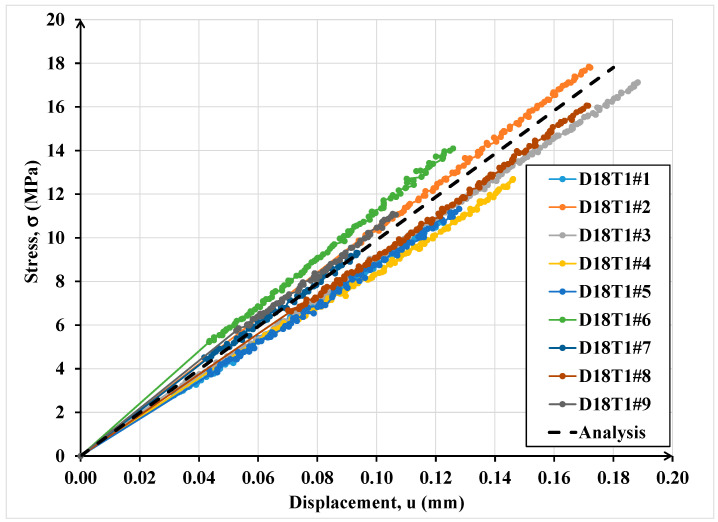
Comparison between numerical and experimental results for the cylindrical specimens with diameter equal to 18 mm and adhesive thickness equal to 1 mm.

Figure 14 shows the comparison between the experimental results of the cylindrical joints of 18 mm diameters connected by an adhesive layer of thickness equal to 1 mm.

**Figure 15:**
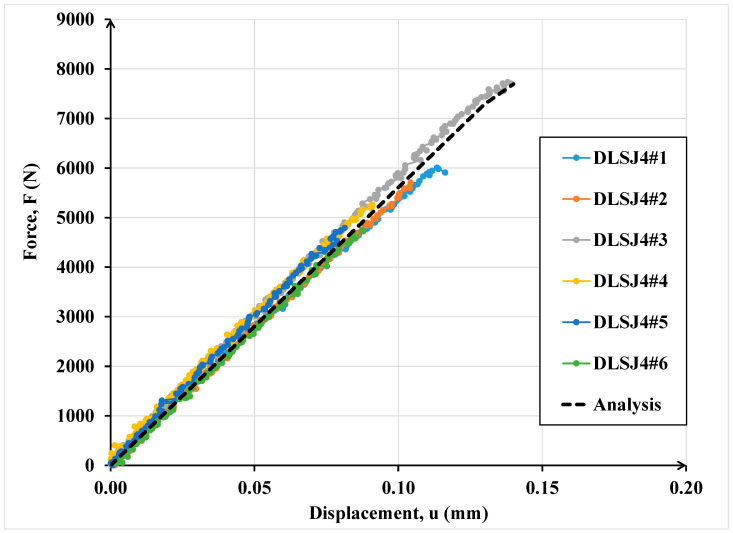
Comparison of numerical and experimental results for the double lap shear adhesive specimens.

In Figure 15, the comparisons between the numerical and experimental results for the double lap shear joints tests are depicted.

As it is possible to note in both cases (Figure 14 and Figure 15), the numerical curves are collocated in the dispersion of experimental evidence. The dispersion of experimental results in terms of ultimate resistance is due to the presence of defects that influence the mechanical behaviour of adhesive connections especially in the case of high thicknesses (double lap shear joints). The numerical data are in good agreement with the experimental ones underlying the power of the interface imperfect model. 

**Figure 16:**
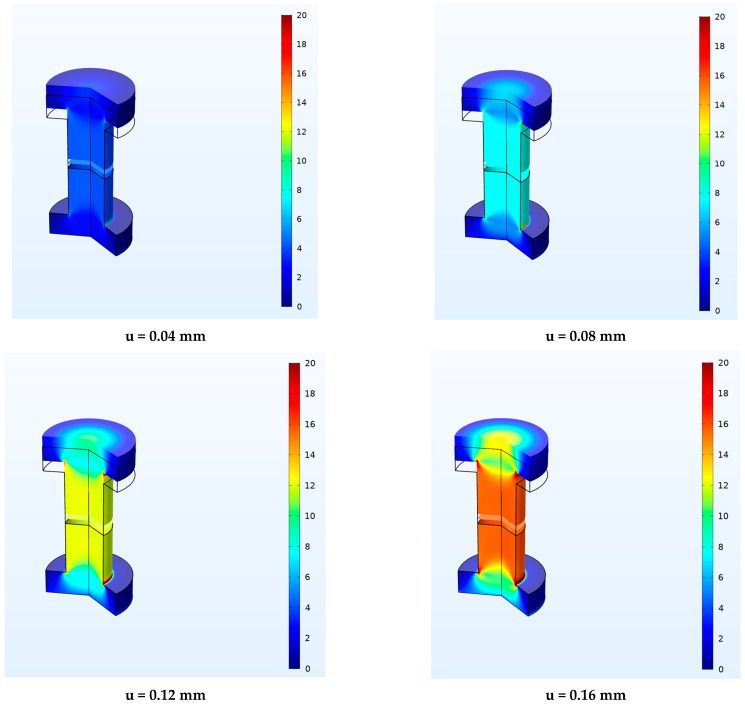
Normal stress distribution in MPa at different displacement levels for cylindrical adhesive joint.

Figure 16 shows the contour plots obtained by the revolution of the 2D numerical model in terms of normal stress at varying of displacement applied, 0.04, 0.08, 0.12 and 0.16 mm, respectively, for the numerical analysis of cylindrical adhesive joint.

**Figure 17:**
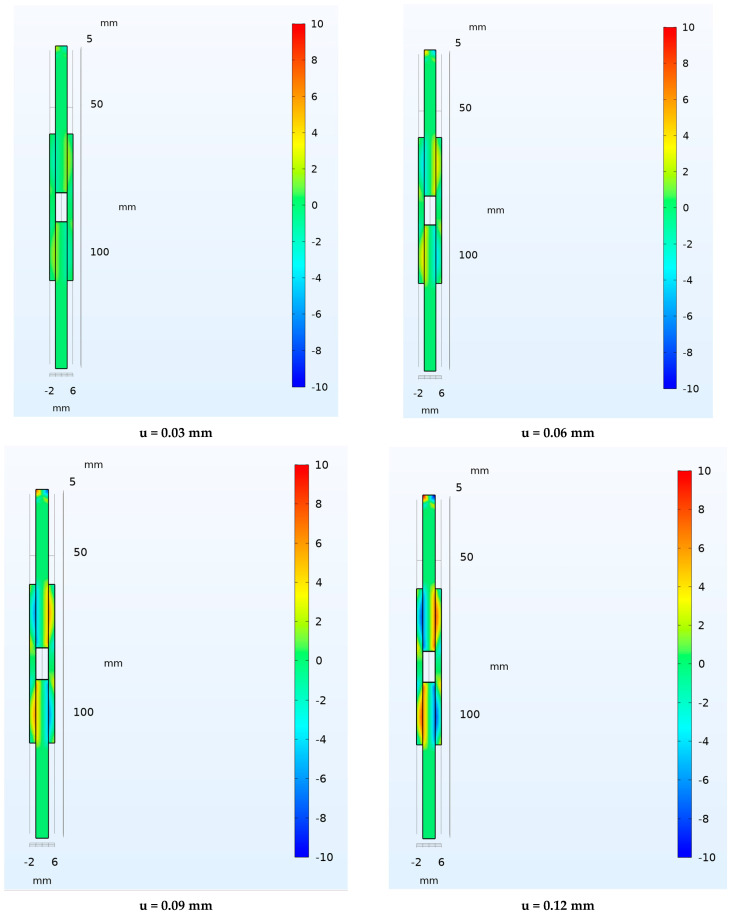
Shear stress distribution in MPa at different displacement levels for double lap shear joint.

Finally, in Figure 17, the contour plots in terms of shear stress at varying of displacement applied, 0.03, 0.06, 0.09 and 0.12 mm, respectively, are presented for the numerical analysis of double lap shear joint.

## 8. Conclusions

An extensive experimental programme has been conducted at the Laboratory of Mechanics and Acoustics in Marseille to evaluate the performance of mechanical adhesive connections for secondary members in structural applications such as offshore installation. In particular, the damage parameters have been evaluated experimentally in order to improve the design process of such connections taking into account the presence of defects or voids. 

The results discussed support the following conclusions:

(1) The volume of the adhesive layer influences the mechanical strength of the joints as experimentally observed.
(2) The damage parameters are functions of the volume of the adhesive layers; a greater quantity of adhesive increases the probability of having defects or voids.
(3) The damage parameters evaluated under Mode I and Mode II loading conditions implemented in the imperfect interface model allow the reproduction of the experimental tests with high accuracy.
(4) The imperfect interface model has been shown to be powerful enough to be used and considered as a design process for adhesive connections.

## Figures and Tables

**Table 1:** Mechanical properties of Sicomin Isobond SR 5030/SD 503x.

Property	Unit Measure	Value
Modulus of Elasticity	N/mm^2^	4500
Tensile strength	N/mm^2^	62
Elongation at break	%	2.9
Shear strength	N/mm^2^	13.4

**Table 2:** Mechanical properties of aluminium cylinder.

Property	Unit Measure	Value
Modulus of Elasticity	N/mm^2^	70,000
Yield strength	N/mm^2^	210
Poisson ratio	-	0.3

**Table 3:** Mechanical properties of steel plate.

Property	Unit Measure	Value
Modulus of Elasticity	N/mm^2^	210,000
Yield’s strength	N/mm^2^	275
Poisson ratio	-	0.3

**Table 4:** Results of adhesive joints with a diameter equal to 18 mm.

Test ID	F_u_	F_u,av_	σ_u_	σ_u,av_	u_max_	u_max,av_	K	K_av_
	(N)	(N)	(MPa)	(MPa)	(mm)	(mm)	(N/mm^3^)	(N/mm^3^)
D18T1#1	2719	3399 ± 781	10.69	13.36 ± 3.07	0.123	0.139 ± 0.03	87	96 ± 9
D18T1#2	4538		17.83		0.172		104	
D18T1#3	4358		17.13		0.188		91	
D18T1#4	3230		12.69		0.146		87	
D18T1#5	2882		11.32		0.128		89	
D18T1#6	3586		14.09		0.126		112	
D18T1#7	2369		9.31		0.094		99	
D18T1#8	4086		16.06		0.171		94	
D18T1#9	2824		11.10		0.106		104	
D18T2.5#1	2877	2794 ± 704	11.31	10.98 ± 2.77	0.142	0.146 ± 0.03	80	75 ± 5
D18T2.5#2	2051		8.06		0.116		70	
D18T2.5#3	3453		13.57		0.179		76	
D18T5#1	2087	2028 ± 953	8.20	10.04 ± 3.59	0.136	0.126 ± 0.05	60	63 ± 4
D18T5#2	3676		14.17		0.207		68	
D18T5#3	1968		7.74		0.117		66	

**Table 5:** Results of adhesive joints with a diameter equal to 14 mm.

Test ID	F_u_	F_u,av_	σ_u_	σ_u,av_	u_max_	u_max,av_	K	K_av_
	(N)	(N)	(MPa)	(MPa)	(mm)	(mm)	(N/mm^3^)	(N/mm^3^)
D14T1#1	2515	1770 ± 645	16.34	11.50 ± 4.19	0.139	0.103 ± 0.03	117	111 ± 9
D14T1#2	1412		9.17		0.091		101	
D14T1#3	1384		8.99		0.078		116	
D14T2.5#1	1915	1703 ± 326	12.44	11.06 ± 2.12	0.106	0.100 ± 0.02	118	111 ± 21
D14T2.5#2	1927		12.52		0.112		112	
D14T2.5#3	1718		11.16		0.076		147	
D14T2.5#4	1283		8.34		0.095		88	
D14T2.5#5	1326		8.61		0.090		96	
D14T2.5#6	2046		13.29		0.124		107	
D14T5#1	1158	1261 ± 228	7.52	8.95 ± 1.48	0.062	0.092 ± 0.03	122	97 ± 25
D14T5#2	1613		10.48		0.118		89	
D14T5#3	1365		8.87		0.122		73	

**Table 6:** Results of adhesive joints with a diameter equal to 10 mm.

Test ID	F_u_	F_u,av_	σ_u_	σ_u,av_	u_max_	u_max,av_	K	K_av_
	(N)	(N)	(MPa)	(MPa)	(mm)	(mm)	(N/mm^3^)	(N/mm^3^)
D10T1#1	875	875	11.15	11.15	0.079	0.079	141	141
D10T2.5#1	648	764 ± 293	8.25	10.72 ± 3.73	0.059	0.076 ± 0.04	140	133 ± 34
D10T2.5#2	568		7.23		0.053		136	
D10T2.5#3	1207		15.37		0.139		111	
D10T2.5#4	944		12.02		0.063		192	
D10T5#1	517	763 ± 241	6.59	9.72 ± 3.07	0.050	0.078 ± 0.02	132	126 ± 17
D10T5#2	768		9.77		0.067		146	
D10T5#3	811		10.33		0.082		126	
D10T5#4	584		7.44		0.074		100	
D10T5#5	1135		14.45		0.115		126	

**Table 7:** Experimental results of double lap shear joints.

Test ID	F_u_	F_u,av_	τ_m_	τ_m,av_	u_max_	u_max,av_	K	K_av_
	(N)	(N)	(MPa)	(MPa)	(mm)	(mm)	(N/mm^3^)	(N/mm^3^)
DLSJ4#1	6010	5780 ± 1014	5.78	5.49 ± 0.97	0.113	0.103 ± 0.02	51	54 ± 2
DLSJ4#2	5707		5.49		0.104		53	
DLSJ4#3	7735		7.44		0.139		53	
DLSJ4#4	5257		5.06		0.091		56	
DLSJ4#5	4793		4.61		0.081		57	
DLSJ4#6	4744		4.56		0.087		52	

**Table 8:** Comparison between the initial damage length evaluated by means of mathematical formulation for Mode I and II.

(a) Mode I				
Thickness	Surface	Volume	Porosity Rate	Initial Damage Length
t (mm)	S (mm^2^)	V (mm^3^)	ρ(l_0_)	l_0_ (mm)
1	254	254	9.91	13.61
	154	154	8.58	10.97
	79	79	6.77	8.10
2.5	254	636	5.08	14.79
	154	385	3.43	10.97
	79	196	2.86	8.25
5	254	3185	1.21	15.66
	154	1924	0.78	11.47
	79	982	0.51	8.41
**(b) Mode II**				
**Thickness**	**Surface**	**Volume**	**Porosity Rate**	**Initial Damage Length**
**t (mm)**	**S (mm^2^)**	**V (mm^3^)**	**ρ(l_0_)**	**l_0_ (mm)**
4	460	1840	3.70	18.94

## Data Availability

The data required to reproduce these findings are all reported in the paper.
